# Successes and failures of sixty years of vector control in French Guiana: what is the next step?

**DOI:** 10.1590/0074-02760170398

**Published:** 2018-03-12

**Authors:** Yanouk Epelboin, Sarah C Chaney, Amandine Guidez, Nausicaa Habchi-Hanriot, Stanislas Talaga, Lanjiao Wang, Isabelle Dusfour

**Affiliations:** 1 Institut Pasteur de la Guyane, Unit Vector Adaptation and Control, Vectopôle Amazonien Emile Abonnenc, Cayenne, French Guiana; 2 Independent Entomologist, Buenos Aires, Argentina

**Keywords:** French Guiana, vector control, insecticide resistance, Anopheles darlingi, Aedes aegypti

## Abstract

Since the 1940s, French Guiana has implemented vector control to contain or eliminate malaria, yellow fever, and, recently, dengue, chikungunya, and Zika. Over time, strategies have evolved depending on the location, efficacy of the methods, development of insecticide resistance, and advances in vector control techniques. This review summarises the history of vector control in French Guiana by reporting the records found in the private archives of the Institute Pasteur in French Guiana and those accessible in libraries worldwide. This publication highlights successes and failures in vector control and identifies the constraints and expectations for vector control in this French overseas territory in the Americas.

French Guiana is located in the northeastern part of the South American continent, sharing a border with Surinam on the West and Brazil on the East (Fig. 1). Colonised in the early 17th century, it became a French department in 1946. The territory was subject to the slave trade until 1848, and was then ruled by a penal colony system until 1946. Ninety percent of the department is covered by the Amazon Rainforest, with human centres of habitation being concentrated on the coast, with a few villages spread along the inland rivers. Due to its geographic position, its immigration history and attractiveness of the European administration, multiple ethnicities shape the Guianese population. As a French department in the Americas, European directives are enforced, including public health standards and pesticide use. Nevertheless, the context of this territory is unique, with a steady immigration rate, an increasing population, and the continuous creation of new human settlements that do not comply with sanitation and water regulations. About 15% of the population was estimated to live without access to drinking water in 2007 (Mansotte et al. 2010a). Over the last several decades, the human population has increased, leading to extensive and sprawling urbanisation. This lack of infrastructure has been closely associated with the production of urban mosquitoes, despite continuous efforts to regulate and remove mosquito breeding sites.

**Fig. 1:**
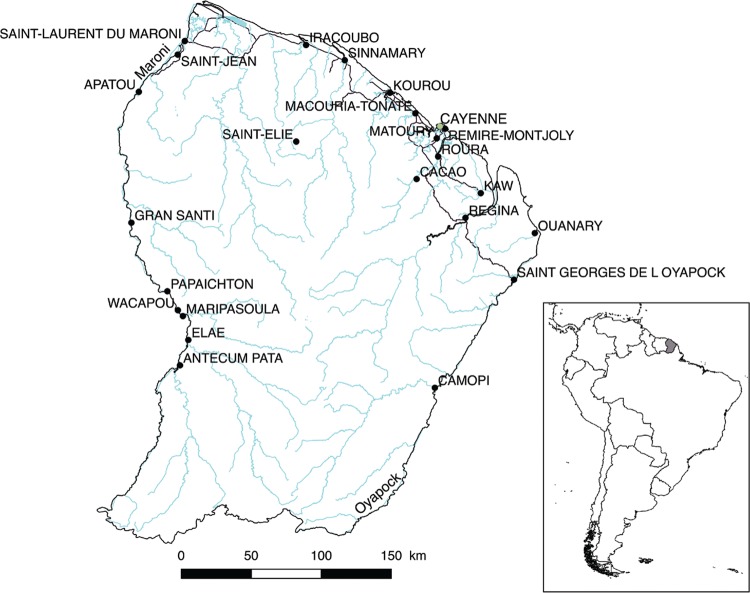
: map of French Guiana with the main human settlements and main towns. Rivers and current roads are shown.

Over the last century, yellow fever (YF), malaria, and dengue have successively threatened the development of French Guiana. YF and dengue are caused by viruses belonging to the Flavivirus genus, and are only transmitted by the mosquito *Aedes aegypti* in the human settlements of French Guiana. The YF virus (YFV) is also maintained in a forest cycle between non-human primates and mosquitoes, such as *Haemagogus* sp. and *Sabethes* sp., which are distributed throughout the Amazon Basin, including French Guiana (Floch 1950, Pajot et al. 1985, de Thoisy et al. 2004, Talaga et al. 2015). Regular outbreaks of YF were confirmed in human settlements from the 18th century to 1902 (Floch 1952a). Vaccination campaigns and eradication programs started in 1949 (Floch 1950), resulting in the disappearance of *Ae. aegypti* for more than 10 years. In addition, while Floch first reported dengue-like epidemics in the early 1940s, regular outbreaks only occurred again after the reintroduction of *Ae. aegypti* in 1963. These epidemics continue to increase in frequency, intensity, and severity (Reynes 1996, Quénel et al. 2011, L’Azou et al. 2014). In 1951, no more cases of YF were recorded. Consequently, malaria remains one of the main public health burdens in French Guiana. Transmitted by *Anopheles* mosquito species, especially *Anopheles darlingi*, the permanent transmission of *Plasmodium* parasites was restricted to inland and bordering areas after the eradication campaign in the 1950s.

Vector-born disease control in French Guiana has been primarily achieved through vector control measures, in addition to vaccines (YF) and chemoprophylaxis (malaria). These vector control strategies have largely relied on insecticide application as the first line of defence to control mosquito populations over many decades. Larval control and, especially, non-chemical source reduction techniques were poorly utilised historically, even though these methods have expanded over time. Few records with limited details on non-chemical larval control were retrieved from the historic reports for this investigation.

Four main families of insecticide compounds are used worldwide: carbamate (CA), organochlorine (OC), organophosphate (OP), and pyrethroid (PY) insecticides. The PY chemical family is described as having low toxicity on humans. Insecticidal molecules belonging to these four families target different ion channels or enzymes to interrupt the proper functioning of the insect nervous system (Liu 2015). Mutations in the amino-acid sequence of binding site of the insecticide target as well as metabolic changes, lead to frequent resistance in mosquito populations (Liu 2015). After decades of insecticide pressure, mosquito populations have become resistant to multiple chemical insecticide families, compromising the effectiveness of chemical-based control (Ranson et al. 2010, Ranson & Lissenden 2016). Today, there is an urgency to develop alternative control methods, including novel insecticides, to better manage resistance and maintain effective tools for fighting vector-borne diseases.

Since 1940, the Institute Pasteur of French Guiana has focused on studying infectious diseases to support and improve their control. Co-authors accessed the archives of the “Institut Pasteur de la Guyane” to review reports and papers since 1949, with the aim to improve our understanding of what successes and failures were encountered by our predecessors, and to discuss what could be done to improve the control of current vectors in the context of an increasing vector-borne disease threat. The entire library archives in our possession were reviewed for articles and data to contribute to a comprehensive picture of the history of vector control in French Guiana.

*Main human pathogen vectors of French Guiana in 1949* - *Ae. aegypti* originated from Africa and, most likely, colonised the Americas during the period of the human slave trade during the 17th century (Soper 1967, Powell & Tabachnick 2013). First reported in the city of Cayenne in 1902 (Neveu-Lemaire 1902), this mosquito was suspected to occur in French Guiana since the 18th century, due to recurrent YF outbreaks, the last of which was documented in 1902 (Fouque 1998). In 1949, *Ae. aegypti* was distributed in all human settlements along the coastal area, as well as inland, in places such Saint Elie, Saül, and Wacapou (upper Maroni, Maripasoula area) (Floch 1950 , Floch & Abonnenc 1951) (Fig. 1). No records reported the species along the Oyapock River. The behaviour of *Ae. aegypti* is strongly related to human settlements: endophilic, anthropophilic, and anthropogenic breeding sites are characteristic of this species, with a total absence outside of human settlements.

*An. darlingi* was first recorded in French Guiana in 1934 by Senevet (Floch 1951). In 1943, a *Plasmodium* infection rate of 1.2% among 542 specimens was recorded. This infection coincided with a peak in malaria cases, with mosquito density incriminating this species as a major malaria vector in the territory (Floch & Abonnenc 1943a). These observations were consistent with those made in Venezuela, Brazil, and British Guyana (Townsend 1934, d, de Bezerra 1936, Gabaldon 1939, Giglioli 1939). This species is also characterised by high anthropophilic feeding and has variable ecology and biting hours (Floch 1950). The species was distributed throughout the whole of French Guiana, but was mainly found inland in forest and savannah areas, rather than on the coast. No specimens were collected in downtown Cayenne, a location considered as too windy and too dry for this species (Floch 1950). Even though the forest is the original environment, the species proliferated and became endophilic and anthropophilic in inland human settlements (Floch 1950, 1956). Some *An. darlingi* populations might also exhibit diurnal behaviours, particularly in forest settlements and gold mines (Floch & Abonnenc 1943a, Floch 1950).

*Anopheles aquasalis* was the main *Anopheles* species collected along the coast. First reported in French Guiana by Thézé in 1916 (as *Cellia albimana*), it was considered a putative malaria vector in Cayenne where *An. darlingi* was not found (Floch & Abonnenc 1943b). While considered an inefficient vector, experimental infection demonstrated its vector competence for *Plasmodium* sp., making it a secondary vector in the coastal area (Floch & Abonnenc 1943b). By 1949, no local transmission was attributed to this vector in French Guiana, in contrast to other countries in South America (Floch & Abonnenc 1943c). The species is both anthropophilic and zoophilic, and exhibits exophilic behaviour.

*Culex quinquefasciatus* was identified as one vector of *Wuchereria bancrofti* in French Guiana in 1945 (Floch & de Lajudie 1945). The species was first described in French Guiana in 1918 by Leger (Floch 1950). The species lives in human environments, especially along coastal areas, and has anthropophilic behaviour. However, this species was not directly targeted by the eradication campaign and was of decreasing interest over the years.

*1949-1959 - The first successes of the YF and malaria eradication campaigns* - Starting in May 1949 in French Guiana, the YF and malaria eradication program aimed to eradicate both diseases by treating or vaccinating patients and eliminating the vectors, *Ae. aegypti* and *Anopheles* species, respectively. The use of chemical compounds was the central component of vector control during this period, through the indoor residual spraying (IRS) of Dichlorodiphenyltrichloroethane (DDT) at 1.5-2 g/m^2^. This campaign started successfully in the city of Cayenne and was extended to all human settlements in French Guiana in the following years (Floch 1950, 1952b, 1953, 1954a, b, 1955a) (Fig. 1).

Control measures against larval stages were not often used, as breeding sites were large and adult control worked remarkably well. Some reports mentioned the drainage of water bodies to protect against *Cx. quinquefasciatus* and *Anopheles* sp. (Floch 1950). Gamma-HCH was occasionally applied on *Cx. quinquefasciatus* breeding sites, as was DDT against *Anopheles* breeding habitat (Floch 1952c, 1954c). The efficacy of local larvivorous fishes was also explored as an alternative tool against *Cx. quinquefasciatus* (Floch 1954c). Actions against the larval stages of *Ae. aegypti* were described as being difficult, even impossible, to deploy.

By 1951, no *Ae. aegypti* specimens were found in French Guiana (Floch 1951). Urban YF was no longer considered a threat. In 1955 and 1956, *Ae. aegypti* was collected in Saint Laurent du Maroni at the Surinam border, but no established colony was observed (Floch 1965a).

In the meantime, malaria cases decreased significantly across the territories along with the density of *An. darlingi*. In 1954, malaria had decreased by 98.8% and pernicious malaria disappeared. Consequently, the economy and quality of life were significantly improved, as indicated by an increase in the human population (Floch 1954c). The vector was still reported across the territory, indicating a continued reintroduction of *An. darlingi* from forested areas to domestic environments (Floch 1951, 1954c, d, e). Such movements of mosquito populations compromised the eradication effort, even if *An. darlingi* was still susceptible to DDT residual spraying. *An. aquasalis* populations were less impacted by the IRS campaign, due to their exophilic and less anthropophilic behaviours (Floch 1954e). In 1954, autochthonous malaria cases were reported in the Cayenne area, following the arrival of immigrants from Saint Lucia. In the absence of any *An. darlingi* specimens, *An. aquasalis* was incriminated (Floch 1955b, d, 1956). Floch hypothesised that the imported strain of *P. falciparum* parasites was adapted to transmission by *An. aquasalis*, the main vector in Saint Lucia, in contrast to the strain that had been circulating in French Guiana. In 1958, *An. aquasalis* remained susceptible to DDT, HCH, and dieldrin according to larval tests performed using Brown protocols (Floch & Fauran 1958)

Supplementary data (Table), Fig. 2]. This secondary vector had rarely been of concern for malaria transmission, and its control was more difficult, due to its behaviour. Therefore, control strategies remained focused on *An. darlingi*.

**Fig. 2:**
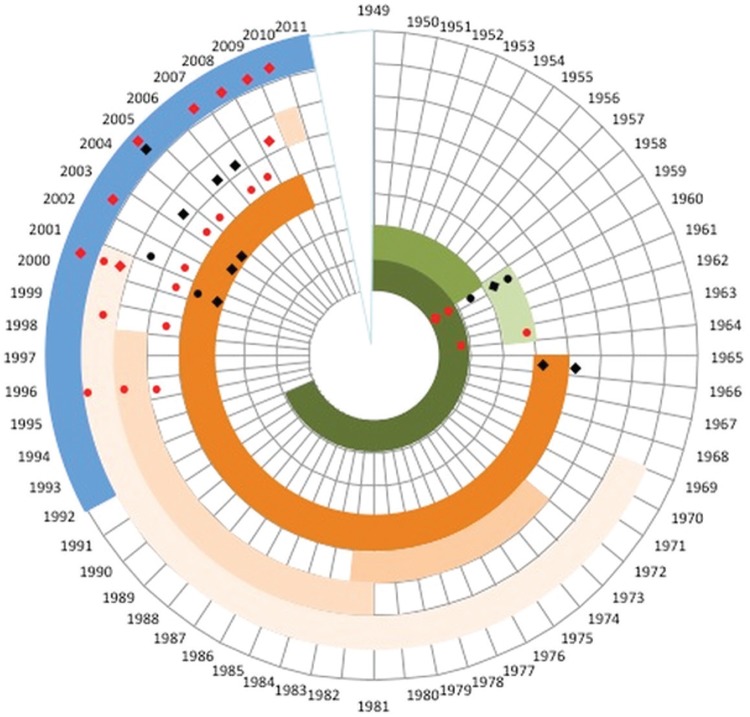
: chronology of insecticide use in French Guiana and bioassays of *Aedes aegypti*. Green represents organochlorines DDT, HCH-Gamma, and dieldrin from dark to light; orange represents organophosphorus: malathion, fenthion, fenitrothion, and temephos from dark to light; blue represents deltamethrin. Squares represent adult tests, circles represent larval testing, dark is for susceptible and red for resistant based on protocols published by World Health Organization over the years.

The first vector control campaigns in 1949 also affected *Cx. quinquefasciatus*. This mosquito disappeared from detection, leading the team in charge of the program to assume that it had been eradicated. However, *Cx. quinquefasciatus* returned the next year and was suspected to be resistant to DDT. The use of gamma-HCH to treat peri-domestic structures (120-150 mg/m^2^; outdoor toilets, henhouse, hovel) and breeding sites, along with the mechanical removal of larval sites, maintained low population densities. Using Brown protocols, populations with resistance to gamma-HCH and dieldrin were detected in Cayenne in 1958. A loss of susceptibility to DDT was also recorded during these tests, confirming the field observation from 1948 (Floch & Fauran 1958). In addition, the installation of water supply systems and covered sewers in Cayenne contributed to an increase in the number of breeding sites. Indeed, during the dry season, drainage was not sufficient and produced stagnant water, which was consequently transformed into seasonal breeding sites for *Cx. quinquefasciatus* (Floch 1951, 19, 1956).

*1959-1963 - Reintroduction of DDT resistant Ae. aegypti populations: a turning-point for vector control strategies* - In 1959, *Ae. aegypti* recolonised French Guiana along the coast from Saint Laurent to Cayenne. Insecticide resistance testing on larvae and adults revealed DDT resistance, but full susceptibility to Gamma-HCH and dieldrin (Fontan & Fauran 1959). Based on this observation, vector control shifted to a campaign of IRS with dieldrin (750 mg/m^2^). The successful campaign started in St Laurent du Maroni and ended in Cayenne. Field teams were not able to collect *Ae. aegypti* after two months of the campaign (Fontan & Fauran 1959).

In 1963, a new introduction was observed in Saint Laurent du Maroni, and spread along road-connected areas and even to non-road-connected areas, such as the Lower Approuague (Régina) and Oyapock (Saint Georges de l’Oyapock) (Floch 1965a). The *Ae. aegypti* resistance test demonstrated that the species was resistant to both DDT and dieldrin [Fig. 2,

Supplementary data (Table)]. An attempt to use dieldrin for IRS was then briefly and inefficiently performed. Another insecticide should have been used to maintain an insecticide-focused vector control program. However, the use of organophosphates, such malathion, fenthion, and fenitrothion was highly controversial at that time, due to their threat to human health, the low persistence of the products, and their high cost (Floch 1964). Larval control was also implemented in 1963, but few results were obtained against *Ae. aegypti*, as expected by Floch. The researcher’s experience demonstrated the poor results of this door-to-door inspection method, despite the high expense and many people needed to implement this type of larval control. At that time, Floch recommended IRS as the main means of controlling YF mosquitoes. In the absence of safe and efficient insecticides, *Ae. aegypti* control remained at a standstill in 1964. Tests of insecticide resistance in YF mosquitoes and the evaluation of product persistence on diverse substrates demonstrated the suitability of malathion for use in indoor residual spraying (Floch et al. 1966).

In the same period, the coast was declared free of *An. darlingi*, but low densities of reinfestation (Floch 1964) occurred over the following years in several places (Floch 1965b). After using DDT for vector control, dry season epidemics ceased and the malaria index dropped to 0.4%, with the majority of cases (94%) occurring in the Oyapock and Maroni river areas.

Subsequently, vector control was not considered as a single strategy. Reports mentioned a first strategy used inland with the objective to target malaria vectors, especially *An. darlingi*, in areas of permanent malaria transmission. The second strategy was applied on the coastal area, and targeted *Ae. aegypti* across its distribution and malaria vectors where malaria transmission occured (Floch 1964, Chaud et al. 2006).

*1963-1991* - *Chemical control dominated by indoor residual sprayed DDT* - Malaria endemic areas were located along the Maroni and Oyapock rivers, on the borders of Suriname and Brazil, respectively. Meanwhile, *Ae. aegypti* was mainly restricted to Cayenne, and was occasionally collected in coastal towns, such as Saint Laurent du Maroni (Cebret & Désiré 1996). Semestrial IRS was performed inland as a control measure, while *Ae. aegypti* and *Anopheles* species were targeted on a case-by-case basis in the coastal areas, where and when either of these cases were detected. Beginning in 1965, formulated DDT (2 mg/m2) or malathion (8%) were used in IRS (Haziza 1973). Over time, *Ae. aegypti* expanded to settlements and towns all along the coast.

Malaria cases increased in the 1970s with the arrival of migrants and the reappearance of *An. darlingi* in several coastal localities (Mouchet et al. 1989). Transmission foci intensified in these areas (Musset et al. 2014), including the areas where only *An. aquasalis* could be incriminated (Silvain 1979, Juminer et al. 1981).

Floch reported the difficulties of implementing larval control and was not convinced of its efficacy (Floch 1964). In the 1970s, larval control for both malaria and arbovirus vectors started to be implemented occasionally by spraying DDT and OP (including temephos, naled, fenthion, and fenitrothion) in larval breeding sites that were positive for the presence of any mosquito species (Haziza 1973, Juminer et al. 1981, Mouchet et al. 1989, Fouque & Laventure 1997, 1998, Claustre et al. 2001). Modalities of spraying were rarely found in reports. Adulticide actions have remained the primary means to control vectors.

*1992-2011* - *Towards synthetic pyrethroids, biological insecticides, and alternative methods to maintain efficacy and environmental safety* - Malaria cases were maintained along the borders and were sporadically reported along the coast. In 1999, the distribution of *Ae. aegypti* expanded further to Maripasoula and Saül. In 2001, Papaïchton, Saint Elie, Ouanary, and Camopi were still free of *Ae. aegypti* (Fouque & Carinci 1996, DDAS/Etat DSP/IPG 2001). Today, the distribution of *Ae. aegypti* still excludes Camopi and the upper Maroni above Elae (Carinci, Unpublished observations).

In 1991, the international ban of DDT was implemented in at least 26 countries worldwide (UNEP/FAO 1991). This molecule was substituted in French Guiana by deltamethrin (25 mg/m^2^), a pyrethroid, for bimestrial or quarterly IRS applications in endemic areas and focal spraying around confirmed malaria cases along coastal areas (Haziza 1973, Pajot et al. 1978, Reynes et al. 1995, Chaud et al. 2006, Musset et al. 2014). To complement these efforts, the use of deltamethrin-impregnated bed nets (15 mg/m^2^), and later Long Lasting Impregnated Nets (LLIN), were reported sporadically since the 1990s (Mouchet et al. 1989, DDAS/Etat DSP/IPG 1993, Claustre et al. 2001, Chaud et al. 2006, Mansotte et al. 2010b, Musset et al. 2014). Bed net distribution, though on a sporadic and voluntary basis, followed the WHO recommendations for the use of Insecticide-Treated Nets (ITNs) as one of the main strategies for malaria control in the Roll Back Malaria program (WHO 1999). At present, ITNs are being distributed in many malaria-endemic regions, and their use has replaced IRS in many countries. LLIN were widely distributed by the Global Fund in Surinam; however, French Guiana, as a French territory, is not eligible for the program (Hiwat et al. 2012). Nevertheless, since 2010, LLIN have been distributed free of charge in malaria endemic areas of French Guiana by the French public health agency (ARS 2015) and are available for purchase in pharmacies. Antilarval treatments are achieved by using *Bacillus thuringiensis* var *israelensis* H14.

Malathion ground-spatial spraying with Ultra-Low Volume application (ULV, 300-400 mL/ha) was first used to complement deltamethrin IRS around detected dengue cases. In the 1990s, malathion ULV spraying, applied weekly or quarterly, fully replaced deltamethrin in Cayenne and Kourou (Juminer et al. 1981). Other organophosphates (such as naled) were occasionally sprayed, as well. Since 1992, malathion spatial spraying was used against *Ae. aegypti* and also for nuisance mosquito control until its prohibition by the European Union in 2009 for environmental and human safety concerns. After this law enforcement, a fenitrothion (OP)-based formulation was sprayed for 1 year, as it demonstrated a higher efficacy in semi-field evaluations than the deltamethrin-based formulation (Dusfour et al. 2011). This molecule was then prohibited and replaced by deltamethrin in 2010, which was the sole molecule authorised and formulated for this use in the European Union and France. At present, deltamethrin-based formulas are used in indoor environments and for spatial spraying against *Ae. aegypti* during epidemics (Dusfour et al. 2011). In addition, publicly available pyrethroid-based insecticides are widely used against flies and other flying insects within households.

In the 1990s, IRS was complemented by the heavy use of organophosphates as larvicides, along with the removal of breeding sites (Fig. 2) (Haziza 1973, Mouchet et al. 1989, Cebret & Désiré 1996, Fouque & Laventure 1997, 19, 1998, Claustre et al. 2001). The chemical treatment of breeding sites declined, due to the development of resistance in *Ae. aegypti* populations and the preference for biological insecticides based on *B. thuringiensis* var *israelensis* H14 (2.5-10 kg/ha) with specific action on dipteran midguts (Fouque & Reynes 1995) (Fig. 2). Temephos use was abandoned in 2000 but was maintained for emergency situations until it was prohibited by law in 2009 in French overseas territories. Today, vector control teams are deployed through routine door-to-door actions to remove breeding sites and prevent larval proliferation (Fouque et al. 1999).

Finally, community education programs based on door-to-door visits and educational programs in schools were mentioned in reports issued in 1993 (DDAS/Etat DSP/IPG 1994).

In 2010, an intervention plan to coordinate surveillance, medical treatment, and vector control efforts during dengue epidemics was defined. This document, called PSAGE-Dengue (“Programme de Surveillance, d’Alerte et de Gestion d’épidémie de dengue”), describes five phases according to the epidemiology, along with the appropriate responses in term of medical diagnosis and vector control.

*Development of insecticide resistance in mosquitoes* - Methods for evaluating insecticide resistance have evolved over time, and have been standardised in terms of generation, age, physiological state, developmental stage, number of mosquitoes, and diagnostic doses, with the aim to compare the results across studies and at a large scale. Throughout the review of historical data, various methodologies for evaluating insecticide resistance have been applied (Brown 1957, WHO 1960, 2016). Consequently, these results cannot be directly compared. We decided to rely on the conclusions of the operators of each evaluation at that time, regarding the levels of resistance, rather than to compare the raw data on percent mortality from each non-standardized evaluation.

Over time, *An. darlingi* has remained susceptible to insecticides used for its control, including DDT, dieldrin, and, recently, deltamethrin (Haziza 1973, Juminer et al. 1981, Rozendaal et al. 1989, Reynes et al. 1995, Duchemin et al. 1996). Deltamethrin resistance was suspected based on impregnated paper WHO protocols from 1995 to 1998 in populations along the Maroni River, but has never been confirmed with high numbers of mosquitoes or validated with good controls (Fouque & Laventure 1998). Some recent publications refer to insecticide resistance in *An. darlingi* populations across the continent (Fonseca-González et al. 2009, Varón et al. 2012, Galardo et al. 2015). While reduced efficacy of insecticides was regularly observed in *Ae. aegypti* or *Cx. quinquefasciatus* populations, *An. darlingi* has never been suspected to be highly resistant. Floch hypothesised that the reintroduction of wild susceptible mosquitoes from the forest into village populations reduced selection for insecticide-resistance (Floch 1955c). This hypothesis is supported by population genetic studies. In Brazil, seasonal gene flow between forested and urban sites was observed in the region of Port Velho (Angêlla et al. 2007). Finally, no insecticide-resistance levels have been tested for other anopheline species in French Guiana.

In comparison, *Ae. aegypti* resistance has been observed and confirmed in French Guiana, even if data were lacking for about 20 years, with the last record to susceptibility being documented in 1972 (Cebret & Désiré 1996) and a brief mention in 1995 (Reynes et al. 1995). After organochlorine resistance, few studies were reported until resistance to fenitrothion, fenthion, and temephos [Fig. 2,

Supplementary data (Table)] (DDAS/Etat DSP/IPG 1995, Girod et al. 2008, Dusfour et al. 2011). Surprisingly, low levels of malathion resistance have been measured (Pocquet et al. 2014). Indeed, a higher level of resistance would be expected after 40 years of malathion use, due to the increasing occurrence of dengue outbreaks since the 1990s. No target site mutation on Ache has ever been reported in *Ae. aegypti* genes, suggesting resistance due to metabolic degradation. These resistance mechanisms and reversal possibilities remain poorly investigated.

It is interesting, however, to note that *Ae. aegypti* was resistant to deltamethrin since the first test in the 2000s, despite the fact that this molecule was not used before that date. However, deltamethrin and DDT have the same molecular target. Thus, we might hypothesize that the continuous pressure and history of DDT use could have maintained some alleles conferring resistance to deltamethrin as well. This cross-resistance was observed in *Ae. aegypti* in Cuba, in which individuals were subjected to deltamethrin selection, exhibiting both DDT and pyrethroid resistance (Rodriguez et al. 2005).

No recent data are available on resistance in *Cx. quinquefasciatus*; however, it would be interesting to investigate this putative vector.

*What’s next?* - Over the last 60 years, insecticidal molecules and application methods replaced one another to maintain a level of vector control efficacy through an empirical approach. The last two decades have resulted in the use of these molecules being restricted in Europe, leaving only pyrethroids available for use in French Guiana. The mosquito species targeted by these control methods have evolved under different pressures. While *An. darlingi* has remained susceptible in almost the entire range of its distribution, *Ae. aegypti* and *Cx. quinquefasciatus* rapidly developed resistance to the authorised insecticide molecules at the early stages of the vector control program in the 1950s-60s. At that time, larval control was rarely considered as an option by public authorities, due to the large breeding sites suitable for anopheline species and the number, variety, and difficulty of access to breeding sites that were favourable to *Ae. aegypti*.

The susceptibility of *An. darlingi* seems to present an opportunity to improve the control of this vector. However, even as the number of malaria cases has decreased, questions and concerns remain about the efficacy of IRS and LLIN methods for malaria control. First, some communities of Amerindians still live in traditional wooden houses without walls called ‘carbet’ even though modern buildings with walls are available for living and administration purposes. The usefulness of IRS in these structures is limited and bed net distribution is widely recommended (Silvain 1979, Juminer et al. 1981, Mouchet et al. 1989, Raccurt 1997). In addition, vector control teams observed an increase in the number of households refusing indoor insecticide sprayings (Mouchet et al. 1989). Furthermore, the occurrence of low levels of *An. darlingi* activity during the day has been well described, with increased activity at dusk and dawn (around 06:30 and 18:30), when people are still outdoors and active (Hiwat et al. 2010, Vezenegho et al. 2016, de Santi et al. 2017). Based on the behavioural plasticity of this species across its distribution range, mosquito behaviour needs to be further assessed in each locality (Hiwat et al. 2010, Hiwat & Bretas 2011). The same holds true for biting preferences. Indeed, both exophily and exophagy in *An. darlingi* are suspected (Pajot et al. 1977, Mouchet et al. 1989, Vezenegho et al. 2016), the opposite of what Floch observed more than 50 years ago (Floch 1950). Therefore, both indoor and outdoor human activities need to be assessed. In addition, other species may be implicated in residual malaria transmission. Finally, larval control based on the use of *Bti* has become more difficult with the presence of large breeding sites and a poor understanding of suitable water sites that facilitate the breeding of putative vectors. All of these components need further investigation to provide evidence for the impact of vector control. Such an assessment would allow vector control teams to adjust their methods and tools in a context of near-elimination in both Surinam and French Guiana (Hiwat et al. 2012, Petit-Sinturel et al. 2016).

Unfortunately, *Ae. aegypti* populations have developed resistance that has impacted the efficacy of chemical control in French Guiana. The first line of control is the removal of breeding sites, along with occasional *Bti* treatments, to reduce the densities of larval stages. Vector control teams from the local authorities, which are in charge of such interventions, also lead educational programs for the general population and in schools to involve communities in a continuous source reduction action. However, a low proportion of the population is fully aware of the necessity to remove potential and active breeding sites, while others are aware but are not taking action (Mieulet & Claeys 2014, Fritzell et al. 2016). The population considers the public authorities responsible for the control of mosquitoes. However, the door-to-door approach requires time, personnel, and regular interventions, as well as the full cooperation of the population to let them enter the yards and houses, and then search for, remove, and/or treat mosquito sources. *Bti* persistence must also be explored with regard to the capacity of vector control teams to regularly monitor efficacy. Community engagement could be useful and necessary to control *Ae. aegypti*, but it takes time, and needs continuous engagement and appropriate education to be successful. Cultural diversity presents additional challenges, with varied knowledge of disease transmission and mosquito life cycle, but is not represented in communication messages. Limited access to a piped water supply is also a challenge for reducing vector sources (Le Tyrant 2013).

During epidemics, deltamethrin remains the only insecticide available to spray to protect against adult mosquitoes in French Guiana; however, a degree of resistance and an impact on chemical control efficacy have recently been demonstrated (Dusfour et al. 2011, 2015, Faucon et al. 2015, 2017). Yet, in the absence of any other alternative, this molecule is (1) sprayed to combat pest mosquitoes, (2) available for use year-round, and (3) present in the long-lasting impregnated bed nets distributed to prevent malaria transmission and during arbovirus epidemics in French Guiana (Mansotte et al. 2010b, ARS 2015). Furthermore, this molecule is used in IRS against malaria. Finally, pyrethroids are also commonly found among household insecticidal sprays, coils, and other publicly available preparations. The resulting pyrethroid pressure is, therefore, high on *Ae. aegypti* and other mosquitoes, even if they are not the intended targets.

French Guiana has recently been subject to severe outbreaks of Zika and chikungunya (Fischer & Staples 2014, Epelboin et al. 2016), but is also facing a lack of options to control disease vectors. While some organisations have considered the burden of vector borne diseases as minor, the importance of the recent Zika outbreak in South America and the frequent re-emergence of yellow fever have uncovered the lack of options available to protect at-risk populations from vector-borne diseases. However, few alternatives are available for use in an epidemic context or as tools in an integrated program of insecticide resistance management. It is urgent to develop or make available effective mosquito control tools for the European Union market. Once again, pyrethroids are the only molecules authorised against adult mosquitoes, with formulated deltamethrin currently being the only adulticide available in French Guiana. In the case of granting special exceptions during outbreaks, other constraints might occur. For instance, malathion spraying was exceptionally implemented for six months during the chikungunya outbreak in 2014; however, the lack of evidence of its effectiveness in containing the epidemics, the mistrust from the Guianese population against this chemical, and released data on the cancerogenic effects caused by this molecule (IARC 2015) pushed local authorities to stop its use.

Beyond the regulatory constraints of introducing a new pesticide to the market or evaluating the suitability of genetically modified or wolbachia-infected mosquitoes, future control plans must be comprehensive to integrate disease vectors and non-vector mosquito control methods, while reducing the use of insecticides, increasing community engagement, and considering environmental changes to prevent the occurrence of breeding sites (WHO 2004, 2012, Bartlett-Healy et al. 2011). In conclusion, any such strategy should be discussed and implemented at a regional scale, since French Guiana practices a policy of insecticide use different to the rest of the Americas, with far more limitations than its neighbouring countries. Despite insecticide policies varying between bordering nations, resistant mosquito populations are not restricted by borders between countries.
