# Supplementary material for: Successes and failures of sixty years of vector control in French Guiana: what is the next step?
Source: Mem Inst Oswaldo Cruz. 2018 Mar 12;113(5):e170398. doi: 10.1590/0074-02760170398 (PMC5851058; doi:10.1590/0074-02760170398)
Supplement: Supplementary file 1 [file 0074-0276-mioc-113-5-e170398-suppl01.pdf]

TABLE

Insecticide resistance status based on insecticide testing of *Aedes aegypti* from 1958 to 2009in several localities in French Guiana

Source of the data	Species	Mosquitoes tested	Collection date or date range	Protocol	Insecticide tested	Number of mosquitoes tested	Collection site	Status
Floch and Fauran (1958)	*Culex quinque-fasciatus*	Wild caught larvae	1958	Brown	DDT	240	Cayenne	loss suscep-tibility
Floch and Fauran (1958)	*C. quinquefas-ciatus*	Wild caught larvae	1958	Brown	HCH	240	Cayenne	Resistant
Floch and Fauran (1958)	*C. quinquefas-ciatus*	Wild caught larvae	1958	Brown	Dieldrin	240	Cayenne	Resistant
Floch and Fauran (1958)	*Anopheles aquasalis*	Wild caught larvae	1958	Brown	DDT	240	Rémire-Montjoly	Susceptible
Floch and Fauran (1958)	*An. aquasalis*	Wild caught larvae	1958	Brown	HCH	240	Rémire-Montjoly	Susceptible
Floch and Fauran (1958)	*An. aquasalis*	Wild caught larvae	1958	Brown	Dieldrin	240	Rémire-Montjoly	Susceptible
Fontan and Fauran (1959)	*Aedes aegypti*	Wild caught larvae	1959	Brown	DDT	345	Saint Laurent du maroni	Resistant
Fontan and Fauran (1959)	*Ae. aegypti*	Wild caught larvae	1959	Brown	HCH	292	Saint Laurent du maroni	Susceptible
Fontan and Fauran (1959)	*Ae. aegypti*	Wild caught larvae	1959	Brown	Dieldrin	235	Saint Laurent du maroni	Susceptible
Fontan and Fauran (1959)	*Ae. aegypti*	adult f1(gorgee)	1959	Brown	DDT	197	Saint Laurent du maroni	Resistant
Fontan and Fauran (1959)	*Ae. aegypti*	adult f1(gorgee)	1959	Brown	Dieldrin	241	Saint Laurent du maroni	Susceptible
Floch (1965a)	*Ae. aegypti*	Wild caught larvae	1963	NA	DDT	NA	Cayenne	Resistant
Floch (1965a)	*Ae. aegypti*	Wild caught larvae	1963	NA	dieldrin	NA	Cayenne	Resistant
Floch et al. (1966)	*Ae. aegypti*	adults	1965	Busvine-Fay	malathion	20	French Guiana	susceptible
Floch et al. (1966)	*Ae. aegypti*	adults	1965	Busvine-Fay	malathion	20	French Guiana	susceptible
Floch et al. (1966)	*Ae. aegypti*	adults	1965	Busvine-Fay	malathion	20	French Guiana	susceptible
Floch et al. (1966)	*Ae. aegypti*	adults	1965	Busvine-Fay	malathion	20	French Guiana	susceptible
Floch et al. (1966)	*Ae. aegypti*	adults	1965	Busvine-Fay	malathion	20	French Guiana	susceptible
Floch et al. (1966)	*Ae. aegypti*	adults	1965	Busvine-Fay	malathion	20	French Guiana	susceptible
Floch et al. (1966)	*Ae. aegypti*	adults	1965	Busvine-Fay	fenthion	20	French Guiana	susceptible
Floch et al. (1966)	*Ae. aegypti*	adults	1965	Busvine-Fay	fenthion	20	French Guiana	susceptible
Floch et al. (1966)	*Ae. aegypti*	adults	1965	Busvine-Fay	fenthion	20	French Guiana	susceptible
Floch et al. (1966)	*Ae. aegypti*	adults	1965	Busvine-Fay	fenthion	20	French Guiana	susceptible
Floch et al. (1966)	*Ae. aegypti*	adults	1965	Busvine-Fay	fenthion	20	French Guiana	susceptible
Cebret and Désiré (1996)	*Ae. aegypti*	NA	1972	NA	NA	NA	French Guiana	susceptible
Annual report, 'Institut Pasteur de la Guyane	*Anopheles darlingi*	adult f0	1995	NA	deltamethrin	NA	French Guiana	susceptible
Annual report, 'Institut Pasteur de la Guyane	*An. darlingi*	adult f1	1996	NA	delta	NA	French Guiana	susceptible

Source of the data	Species	Mosquitoes tested	Collection date or date range	Protocol	Insecticide tested	Number of mosquitoes tested	Collection site	Status
Annual report, 'Institut Pasteur de la Guyane	*Ae. aegypti*	wild-caught larvae	1995	dose-response	Fenitrothion	1200	Cayenne, Rémire-Montjoly, Matoury & Sinnamary	Resistant
Annual report, 'Institut Pasteur de la Guyane	*Ae. aegypti*	wild-caught larvae	1995	dose-response	Fenthion	400	Cayenne, Rémire-Montjoly, Matoury & Sinnamary	Resistant
Annual report, 'Institut Pasteur de la Guyane	*Ae. aegypti*	wild-caught larvae	1995	dose-response	Temephos	1200	Cayenne, Rémire-Montjoly, Matoury & Sinnamary	Resistant
Annual report, 'Institut Pasteur de la Guyane	*Ae. aegypti*	wild-caught larvae	1998	dose-response	Fenitrothion	400	Cayenne, Rémire-Montjoly, Matoury & Sinnamary	Resistant
Annual report, 'Institut Pasteur de la Guyane	*Ae. aegypti*	wild-caught larvae	1998	dose-response	Fenthion (Baytex)	800	Cayenne, Rémire-Montjoly, Matoury & Sinnamary	Resistant
Annual report, 'Institut Pasteur de la Guyane	*Ae. aegypti*	wild-caught larvae	1998	dose-response	Temephos	1200	Cayenne, Rémire-Montjoly, Matoury & Sinnamary	Resistant
Annual report, 'Institut Pasteur de la Guyane	*Ae. aegypti*	adults reared from wild caught larvae	2000	diagnostic dose	Deltamethrin	123	degrad des cannes, Cayenne	Resistant
Annual report, 'Institut Pasteur de la Guyane	*Ae. aegypti*	wild-caught larvae	2000	dose unique	Malathion	100	Rémire-Montjoly	Susceptible
Annual report, 'Institut Pasteur de la Guyane	*Ae. aegypti*	adults reared from wild caught larvae	2000	diagnostic dose	Malathion	97	Rémire-Montjoly	Susceptible
Annual report, 'Institut Pasteur de la Guyane	*Ae. aegypti*	wild-caught larvae	2000	dose unique	Malathion	100	zéphir, Cayenne	Susceptible
Annual report, 'Institut Pasteur de la Guyane	*Ae. aegypti*	adults reared from wild caught larvae	2000	diagnostic dose	Malathion	100	zéphir, Cayenne	Susceptible
Annual report, 'Institut Pasteur de la Guyane	*Ae. aegypti*	wild-caught larvae	2000	diagnostic dose	Temephos	196	degrad des cannes, Cay-enne	Resistant
Annual report, 'Institut Pasteur de la Guyane	*Ae. aegypti*	wild-caught larvae	2000	diagnostic dose	Temephos	92	degrad des cannes, Cay-enne	Resistant
Annual report, 'Institut Pasteur de la Guyane	*Ae. aegypti*	wild-caught larvae	2000	dose-response	Temephos	NA	Lamirande, Matoury	Resistant
Annual report, 'Institut Pasteur de la Guyane	*Ae. aegypti*	wild-caught larvae	2000	dose-response	Temephos	NA	Rémire-Montjoly	Resistant
Annual report, 'Institut Pasteur de la Guyane	*Ae. aegypti*	wild-caught larvae	2000	dose-response	Temephos	NA	Saint Laurent du Maroni	Resistant
Annual report, 'Institut Pasteur de la Guyane	*Ae. aegypti*	wild-caught larvae	2000	dose-response	Temephos	NA	zéphir, Cayenne	Resistant
Annual report, 'Institut Pasteur de la Guyane	*Ae. aegypti*	F1 larvae	2001	dose-response	Temephos	395	Lamirande, Matoury	Resistant
Annual report, 'Institut Pasteur de la Guyane	*Ae. aegypti*	F1 larvae	2001	dose-response	Temephos	NA	Rémire-Montjoly	Resistant

Source of the data	Species	Mosquitoes tested	Collection date or date range	Protocol	Insecticide tested	Number of mosquitoes tested	Collection site	Status
Annual report, 'Institut Pasteur de la Guyane	*Ae. aegypti*	wild-caught larvae	2001	dose-response	Temephos	NA	Saint Laurent du Maroni	Resistant
Annual report, 'Institut Pasteur de la Guyane	*Ae. aegypti*	F2 larvae	2001	dose-response	Temephos	NA	Zéphir, Cayenne	Resistant
Annual report, 'Institut Pasteur de la Guyane	*Ae. aegypti*	wild-caught larvae	2002	diagnostic dose	fenitrothion	54	Matoury	Susceptible
Annual report, 'Institut Pasteur de la Guyane	*Ae. aegypti*	wild-caught larvae	2002	diagnostic dose	fenitrothion	50	Rémire-Montjoly	Susceptible
Annual report, 'Institut Pasteur de la Guyane	*Ae. aegypti*	wild-caught larvae	2002	diagnostic dose	fenitrothion	100	Rémire-Montjoly	Susceptible
Annual report, 'Institut Pasteur de la Guyane	*Ae. aegypti*	wild-caught larvae	2002	diagnostic dose	fenitrothion	100	Macouria	Susceptible
Annual report, 'Institut Pasteur de la Guyane	*Ae. aegypti*	Adults from wild caught larvae	2002	diagnostic dose	deltamethrin	70	Macouria	Resistant
Annual report, 'Institut Pasteur de la Guyane	*Ae. aegypti*	F1 larvae	2003	diagnostic dose	Temephos	194	Matoury	Resistant
Annual report, 'Institut Pasteur de la Guyane	*Ae. aegypti*	F1 larvae	2003	dose unique	bti	100	Matoury	Susceptible
Annual report, 'Institut Pasteur de la Guyane	*Ae. aegypti*	F1 larvae	2003	dose unique	bti	100	Matoury	Susceptible
Annual report, 'Institut Pasteur de la Guyane	*Ae. aegypti*	Adults from wild caught larvae	2003	diagnostic dose	malathion	100	Rémire-Montjoly	Susceptible
Annual report, 'Institut Pasteur de la Guyane	*Ae. aegypti*	F1 Adults	2004	diagnostic dose	deltamethrin	75	Matoury	Resistant
Annual report, 'Institut Pasteur de la Guyane	*Ae. aegypti*	F1 Adults	2004	diagnostic dose	deltamethrin	73	Rémire-Montjoly	Resistant
Annual report, 'Institut Pasteur de la Guyane	*Ae. aegypti*	F1 Adults	2004	diagnostic dose	fenitrothion	100	Matoury	Susceptible
Annual report, 'Institut Pasteur de la Guyane	*Ae. aegypti*	F1 Adults	2004	diagnostic dose	fenitrothion	80	Cayenne	Susceptible
Annual report, 'Institut Pasteur de la Guyane	*Ae. aegypti*	F1 Adults	2004	diagnostic dose	fenitrothion	100	Kourou	Susceptible
Annual report, 'Institut Pasteur de la Guyane	*Ae. aegypti*	F1 Adults	2004	diagnostic dose	fenitrothion	100	Cacao	Susceptible
Annual report, 'Institut Pasteur de la Guyane	*Ae. aegypti*	F1 Adults	2004	diagnostic dose	malathion	100	Saint Jean du Maroni	Susceptible
Annual report, 'Institut Pasteur de la Guyane	*Ae. aegypti*	F1 Adults	2004	diagnostic dose	malathion	100	Régina	Susceptible

Source of the data	Species	Mosquitoes tested	Collection date or date range	Protocol	Insecticide tested	Number of mosquitoes tested	Collection site	Status
Annual report, 'Institut Pasteur de la Guyane	*Ae. aegypti*	F1 Adults	2004	diagnostic dose	deltamethrin	100	Saint Jean du Maroni	Susceptible
Annual report, 'Institut Pasteur de la Guyane	*Ae. aegypti*	F1 larvae	2004	diagnostic dose	Temephos	200	Matoury	Resistant
Annual report, 'Institut Pasteur de la Guyane	*Ae. aegypti*	F1 larvae	2004	diagnostic dose	Temephos	200	Rémire-Montjoly	Resistant
Annual report, 'Institut Pasteur de la Guyane	*Ae. aegypti*	F1 larvae	2004	dose unique	bti	100	Matoury	Susceptible
Annual report, 'Institut Pasteur de la Guyane	*Ae. aegypti*	F1 larvae	2004	dose unique	bti	100	Rémire-Montjoly	Susceptible
Annual report, 'Institut Pasteur de la Guyane	*Ae. aegypti*	F1 larvae	2004	diagnostic dose	Temephos	100	Matoury	Resistant
Annual report, 'Institut Pasteur de la Guyane	*Ae. aegypti*	F1 larvae	2004	diagnostic dose	Temephos	100	Rémire-Montjoly	Resistant
Annual report, 'Institut Pasteur de la Guyane	*Ae. aegypti*	F1 larvae	2004	diagnostic dose	Temephos	100	Cayenne	Resistant
Annual report, 'Institut Pasteur de la Guyane	*Ae. aegypti*	F1 larvae	2004	diagnostic dose	Temephos	100	Kourou	Resistant
Annual report, 'Institut Pasteur de la Guyane	*Ae. aegypti*	F1 larvae	2004	diagnostic dose	Temephos	100	Cacao	Resistant
Annual report, 'Institut Pasteur de la Guyane	*Ae. aegypti*	F1 larvae	2004	dose unique	bti	100	Cacao	Susceptible
Annual report, 'Institut Pasteur de la Guyane	*Ae. aegypti*	F1 larvae	2004	diagnostic dose	Temephos	100	Régina	Resistant
Annual report, 'Institut Pasteur de la Guyane	*Ae. aegypti*	F1 larvae	2004	diagnostic dose	Temephos	100	Saint Jean du Maroni	Resistant
Annual report, 'Institut Pasteur de la Guyane	*Ae. aegypti*	F1 larvae	2004	dose unique	bti	100	Régina	Susceptible
Annual report, 'Institut Pasteur de la Guyane	*Ae. aegypti*	F1 larvae	2004	dose unique	bti	100	Saint Jean du Maroni	Susceptible
Annual report, 'Institut Pasteur de la Guyane	*Ae. aegypti*	wild-caught larvae	2006	diagnostic dose	Temephos	100	degrad des cannes, Ré-mire-Montjoly	Resistant
Annual report, 'Institut Pasteur de la Guyane	*Ae. aegypti*	wild-caught larvae	2006	diagnostic dose	Temephos	100	Cabassou, Cayenne	Resistant
Annual report, 'Institut Pasteur de la Guyane	*Ae. aegypti*	wild-caught larvae	2006	diagnostic dose	Temephos	100	Tarzan, Cayenne	Resistant
Annual report, 'Institut Pasteur de la Guyane	*Ae. aegypti*	wild-caught larvae	2006	diagnostic dose	Temephos	100	Matoury	Resistant

Source of the data	Species	Mosquitoes tested	Collection date or date range	Protocol	Insecticide tested	Number of mosquitoes tested	Collection site	Status
Annual report, 'Institut Pasteur de la Guyane	*Ae. aegypti*	wild-caught larvae	2006	diagnostic dose	Temephos	100	Rémire-Montjoly	Resistant
Annual report, 'Institut Pasteur de la Guyane	*Ae. aegypti*	Adults from wild-caught larvae	2006	diagnostic dose	deltamethrin	97	Tarzan, Cayenne	Resistant
Annual report, 'Institut Pasteur de la Guyane	*Ae. aegypti*	Adults from wild-caught larvae	2006	diagnostic dose	deltamethrin	95	Rémire-Montjoly	Resistant
Annual report, 'Institut Pasteur de la Guyane	*Ae. aegypti*	wild-caught larvae	2006	dose-response	Temephos	NA	degrad des cannes, Ré-mire-Montjoly	Resistant
Annual report, 'Institut Pasteur de la Guyane	*Ae. aegypti*	wild-caught larvae	2006	dose-response	Temephos	NA	Cabassou, Cayenne	Resistant
BASAG 2008 N°5, Girod et al. (2008)	*Ae. aegypti*	wild-caught larvae	2006	diagnostic dose	Temephos	100	Saint Laurent du Maroni	Resistant
BASAG 2008 N°5, Girod et al. (2008)	*Ae. aegypti*	wild-caught larvae	2006	diagnostic dose	Temephos	100	Régina	Resistant
BASAG 2008 N°5, Girod et al. (2008)	*Ae. aegypti*	wild-caught larvae	2006	diagnostic dose	Temephos	100	Mana	Resistant
BASAG 2008 N°5, Girod et al. (2008)	*Ae. aegypti*	wild-caught larvae	2006	diagnostic dose	Temephos	100	Iracoubo	Resistant
BASAG 2008 N°5, Girod et al. (2008)	*Ae. aegypti*	wild-caught larvae	2006	diagnostic dose	Temephos	83	Apatou	Resistant
BASAG 2008 N°5, Girod et al. (2008)	*Ae. aegypti*	Adults from wild-caught larvae	2006	diagnostic dose	deltamethrin	98	Apatou	Resistant
BASAG 2008 N°5, Girod et al. (2008)	*Ae. aegypti*	Adults from wild-caught larvae	2006	diagnostic dose	deltamethrin	100	Régina	Resistant
BASAG 2008 N°5, Girod et al. (2008)	*Ae. aegypti*	Adults from wild-caught larvae	2006	diagnostic dose	deltamethrin	97	Iracoubo	Resistant
BASAG 2008 N°5, Girod et al. (2008)	*Ae. aegypti*	Adults from wild-caught larvae	2006	diagnostic dose	deltamethrin	100	Saint Laurent du Maroni	Resistant
BASAG 2008 N°5, Girod et al. (2008)	*Ae. aegypti*	Adults from wild-caught larvae	2006	diagnostic dose	deltamethrin	100	Javouhey	Resistant
BASAG 2008 N°5, Girod et al. (2008)	*Ae. aegypti*	Adults from wild-caught larvae	2006	diagnostic dose	fenitrothion	100	Apatou	Susceptible
BASAG 2008 N°5, Girod et al. (2008)	*Ae. aegypti*	Adults from wild-caught larvae	2006	diagnostic dose	fenitrothion	100	Régina	Susceptible
BASAG 2008 N°5, Girod et al. (2008)	*Ae. aegypti*	Adults from wild-caught larvae	2006	diagnostic dose	fenitrothion	100	Saint Laurent du Maroni	Susceptible
BASAG 2008 N°5, Girod et al. (2008)	*Ae. aegypti*	Adults from wild-caught larvae	2006	diagnostic dose	fenitrothion	100	Mana	Susceptible

Source of the data	Species	Mosquitoes tested	Collection date or date range	Protocol	Insecticide tested	Number of mosquitoes tested	Collection site	Status
BASAG 2008 N°5, Girod et al. (2008)	*Ae. aegypti*	Adults from wild-caught larvae	2006	diagnostic dose	fenitrothion	100	Iracoubo	Susceptible
BASAG 2008 N°5, Girod et al. (2008)	*Ae. aegypti*	wild-caught larvae	2007	diagnostic dose	Temephos	100	Saint Georges de l'Oyapock	Resistant
BASAG 2008 N°5, Girod et al. (2008)	*Ae. aegypti*	Adults from wild-caught larvae	2007	diagnostic dose	deltamethrin	80	Saint Georges de l'Oyapock	Resistant
BASAG 2008 N°5, Girod et al. (2008)	*Ae. aegypti*	Adults from wild-caught larvae	2007	diagnostic dose	fenitrothion	45	Saint Georges de l'Oyapock	Susceptible
Dusfour et al. (2011)	*Ae. aegypti*	F1 adults	2008	diagnostic dose	deltamethrin	302	Saint Georges de l'Oyapock	Resistant
Dusfour et al. (2011)	*Ae. aegypti*	F1 adults	2009	diagnostic dose	deltamethrin	208	Cayenne	Resistant
Dusfour et al. (2011)	*Ae. aegypti*	F1 adults	2009	diagnostic dose	deltamethrin	197	Kourou	Resistant
